# The lncRNA ZNF295-AS1 alleviates lung squamous cell carcinoma progression by reducing miR-96-5p and inhibiting cancer cell invasiveness

**DOI:** 10.1080/15476286.2026.2669707

**Published:** 2026-05-14

**Authors:** Yan Huang, Dong Yan, Zhenhua Hao, Xunqing Ni

**Affiliations:** a Department of Respiratory Medicine, The First Affiliated Hospital of Yangtze University, Jingzhou, China; b Department of Cardiothoracic Surgery, The First People’s Hospital of Zhengzhou, Zhengzhou, China; c Department of Cardiothoracic Surgery, Huashan Hospital, Fudan University, Shanghai, China; d Department of Respiratory and Critical Care Medicine, Linyi Hospital of Traditional Chinese Medicine, Linyi, China

**Keywords:** ZNF295-AS1, miR-96-5p, lung squamous cell carcinoma, cell migration, cell invasion

## Abstract

Lung squamous cell carcinoma (LUSC) is highly invasive, and patients with advanced disease generally have a poor prognosis. ZNF295-AS1 is abnormally expressed in lung cancer. This study aims to investigate the prognostic value of ZNF295-AS1 in LUSC and its potential regulatory mechanisms. This study enrolled 116 patients with lung upper-segment carcinoma (LUSC) and obtained LUSC tissue and adjacent normal tissue during surgery. RT-qPCR was used to assess the expression levels of ZNF295-AS1 and miR-96-5p in tissues and cell lines. Cox proportional hazards analysis identified independent factors affecting LUSC prognosis. CCK-8 and Transwell assays assessed cellular proliferation, invasion, and migration capabilities, respectively. DLR validated the relationship between ZNF295-AS1 and miR-96-5p. Compared with normal tissue and BEAS-2B cells, ZNF295-AS1 is significantly downregulated in LUSC tissue and cells. ZNF295-AS1 negatively regulates miR-96-5p level by binding to it as a target. ZNF295-AS1 serves as a protective factor influencing LUSC prognosis. Furthermore, elevating ZNF295-AS1 levels reduces miR-96-5p expression in cells, thereby diminishing the proliferation, migration, and invasion capabilities of LUSC cell lines and mitigating LUSC progression. ZNF295-AS1 demonstrates significant prognostic value in clinical settings for LUSC and holds promise as a novel prognostic biomarker. ZNF295-AS1 acts as a protective factor against LUSC. Mechanistically, ZNF295-AS1 alleviates LUSC progression and improves patient prognosis by regulating miR-96-5p levels.

## Introduction

Lung cancer (LC) ranks among the most prevalent and deadly malignant tumours globally, primarily originating from bronchial mucosal or alveolar epithelial cells [1]. It is classified into two subtypes: small cell carcinoma and non-small cell carcinoma, with the latter accounting for the vast majority of cases [2]. Lung squamous cell carcinoma (LUSC) constitutes 30% of all non-small cell lung cancers (NSCLC) and is strongly associated with smoking [3,4]. Despite advances in treatments such as surgery, radiotherapy, and chemotherapy, the long-term prognosis for LUSC patients remains unsatisfactory. Five-year survival rates persistently hover at low levels, particularly in advanced-stage patients where therapeutic efficacy is severely limited [5]. Therefore, identifying novel biomarkers at the molecular level that can accurately predict the prognosis of LUSC is crucial for improving patient survival outcomes.

In recent years, long noncoding RNA (lncRNA) has been demonstrated to play a pivotal role in the initiation and progression of LC as a key epigenetic regulator, exhibiting significant potential as an ideal prognostic biomarker and therapeutic target [6,7]. Among these, lncRNA ZNF295-AS1 has been reported to be notably downregulated in lung adenocarcinoma [8], and its expression is also downregulated in gefitinib-resistant lung adenocarcinoma (LUAD) cell lines [9]. However, its specific function and mechanisms in LUSC remain unclear.

Previous studies suggest that one of the core functional mechanisms of lncRNAs lies in acting as competitive endogenous RNAs (ceRNAs) [10,11]. Through a ‘sponge effect’, they interact with microRNAs (miRNAs), thereby relieving miRNA suppression on their downstream target genes [12,13]. Using the lncRNA SNP2 database, we identified a potential binding site between ZNF295-AS1 and miR-96-5p. Notably, Ding et al. investigated ceRNA networks in LUSC patients, where miR-96-5p was implicated [14]. Furthermore, miR-96-5p has been identified as an oncogene in multiple studies of lung cancer, including lung adenocarcinoma and NSCLC [15,16]. More importantly, the expression and regulatory significance of miR-96-5p in LUSC patients remain under-explored. Thus, we propose a scientific hypothesis: Could ZNF295-AS1 participate in regulating LUSC progression by targeting and binding miR-96-5p? Currently, the mechanism underlying this potential regulatory axis in LUSC remains unexplored. Based on this, the present study aims to investigate the expression of the ZNF295-AS1/miR-96-5p molecular axis in LUSC and its specific regulatory mechanisms, to provide new theoretical foundations and potential therapeutic targets for the prognosis and treatment of LUSC.

## Materials and methods

### Inclusion of patients

This study included 116 patients with LUSC who were diagnosed and treated at The First People’s Hospital of Zhengzhou between November 2018 and January 2020. Inclusion criteria: a) meeting diagnostic criteria for LUSC; b) complete clinical records. Exclusion criteria: a) history of other malignancies; b) severe infection; c) cardiovascular or psychiatric disorders. All LUSC patients underwent treatment at our hospital, where tumour tissue and a portion of adjacent normal tissue were resected during surgery as a control. Patients were followed up for 5 years via telephone or outpatient visits to record survival status and time to recurrence.

The present study received approval from the ethics committee of The First People’s Hospital of Zhengzhou, with written informed consent obtained from all patients.

### Cell culture and transfected

Normal human bronchial epithelial cells BEAS-2B and four LUSC cell lines SK-MES-1, HCC95, H520, and H226 were purchased from the Shanghai Academy of Sciences Cell Bank (Shanghai, China). All five cell lines were cultivated in RPMI 1640 medium (Gibco, USA), which was enriched with 10% foetal bovine serum (FBS; Gibco, USA). Incubators were maintained at a temperature of 37°C, with a carbon dioxide concentration of 5%.

Cells destined for transfection were seeded into well plates. When cell confluence reached 70–80%, procedures were performed according to the Lipofectamine 3000 kit (Invitrogen, USA) instructions. Briefly, the ZNF295-AS1 overexpression plasmid or its empty vector control, and the miR-96-5p mimic or its negative control (GenePharma, China), with diluted transfection reagent. Incubate at room temperature to form stable DNA-liposome complexes, then uniformly add to the cell culture medium. Replace with fresh complete medium 8 hours post-transfection for continued culture. Collect cells 48 hours post-transfection for subsequent experiments.

### Real-time quantitative reverse transcription PCR (RT-qPCR)

Total RNA was extracted from tissue and cell samples using TRIzol reagent (Invitrogen, USA). The total RNA was reverse transcribed into cDNA by means of the HiScript® III 1st Strand cDNA Synthesis Kit (+gDNA wiper) (Vazyme, Nanjing, China). PCR amplification was performed using the SYBR Premix Ex Taq II Reagent Kit (Takara, Beijing, China) in a 20 μL reaction system. The reaction program was set as follows: 30 s pre-denaturation at 95°C, followed by 40 cycles of amplification with each cycle consisting of 5s denaturation at 95°C, 30s annealing at 56°C, and 30s extension at 72°C. Glyceraldehyde-3-phosphate dehydrogenase (GAPDH) was designated as the internal control gene for ZNF295-AS1, and U6 was assigned this role for miR-96-5p. The relative levels of ZNF295-AS1 and miR-96-5p were calculated using the 2^−ΔΔCt^ method.

### Cell proliferation assay

LUSC cells were resuspended after digestion. Following counting, they were uniformly seeded into a 96-well plate at a density of approximately 1 × 10^4^ cells/well. The plate was then returned to the cell culture incubator for continued culture. Cultures were terminated at 24, 48, and 72 hours, and their absorbance values of each well were measured using the Cell Counting Kit-8 (CCK-8; Solarbio, China). At each designated time point, 10 μL of CCK-8 reagent was added to each well. Subsequently, the plates were subjected to an incubation process within a 37°C incubator, in conditions of darkness, for a duration of two hours. Subsequent to the incubation period, the absorbances were measured at a wavelength of 450 nm, employing a microplate reader (BioTek Synergy H1, USA). A curve was plotted with time on the x-axis and absorbance values on the y-axis.

### Transwell assay

Cell migration and invasion experiments primarily followed the methodology described by Xu [17] and Dang [18]: Migration Assay: Resuspend SK-MES-1 and H520 cells. Add 200 μL of cell suspension (approximately 5 × 10^4^ cells) to the upper chamber of the Transwell. The lower chamber should receive 500 μL of complete medium containing 10% FBS. Incubate at 37°C for 48 hours. Fix cells migrating into the lower chamber with formaldehyde and stain with crystal violet. Five random fields of view are selected for counting under a microscope. Invasion Assay: Prior to cell seeding, uniformly coat the upper chamber of the Transwell with diluted Matrigel matrix (BD Biosciences, USA) gel. Follow subsequent steps as described in the migration assay protocol.

### Bioinformatics analysis

The binding site between ZNF295-AS1 and miR-96-5p was predicted using the LncRNA SNP2 database (https://guolab.wchscu.cn/lncRNASNP/#!/). The downstream target genes of miR-96-5p were predicted using three databases: TargetScan (https://www.targetscan.org/vert_72/), miRDB (https://mirdb.org/) and miRWalk (http://mirwalk.umm.uni-heidelberg.de/). The intersection of the three sets of predictions was selected.

The high-confidence target gene set was submitted to the STRING database (https://cn.string-db.org/) to assess protein-protein interactions. A composite score threshold of >0.4 was set for inclusion. Genes with node degrees greater than zero in this network were extracted and defined as core target genes for subsequent functional enrichment analysis. Gene Ontology (GO) and Kyoto Encyclopedia of Genes and Genomes (KEGG) pathway enrichment analyses were performed. GO terms encompassing ‘biological process’, ‘cellular component’, and ‘molecular function’, along with KEGG pathways exhibiting Benjamini-Hochberg corrected *p*-values < 0.05, were considered statistically significant.

### Dual-luciferase reporter (DLR) assay

Based on the predicted binding sites in the database, we synthesized a wild-type fragment of ZNF295-AS1 (ZNF295-AS1-WT) containing the miR-96-5p binding site and constructed the ZNF295-AS1-WT reporter plasmid. Simultaneously, we mutated the sequence at the binding site to generate a mutant fragment of ZNF295-AS1 (ZNF295-AS1-MUT) plasmid. Subsequently, SK-MES-1 cells were put in well plates. The recombinant plasmids were co-transfected into cells with miR-96-5p mimics, inhibitors, or their negative controls. Following a 48 h transfection period, the harvesting of the cells and the subsequent measurement of luciferase activity was conducted using a DLR assay system.

### Statistical analysis

All results in this study are presented as mean ± standard deviation (SD). Statistical analysis of all data was performed using SPSS 23.0 (IBM, USA) or GraphPad Prism 9.0 (GraphPad Software, USA). Student’s t-test was employed to compare differences between two groups of continuous variables; one-way analysis of variance (ANOVA) was used when comparing multiple groups. The differences were considered to be statistically significant if the *p*-value was less than 0.05. Kaplan-Meier curves were used to analyse survival times in LUSC patients. We employed Cox regression analysis to determine which factors influence the survival probability of patients with LUSC.

## Results

### Expression level of ZNF295-AS1 and its clinical significance

**Figure 1.**
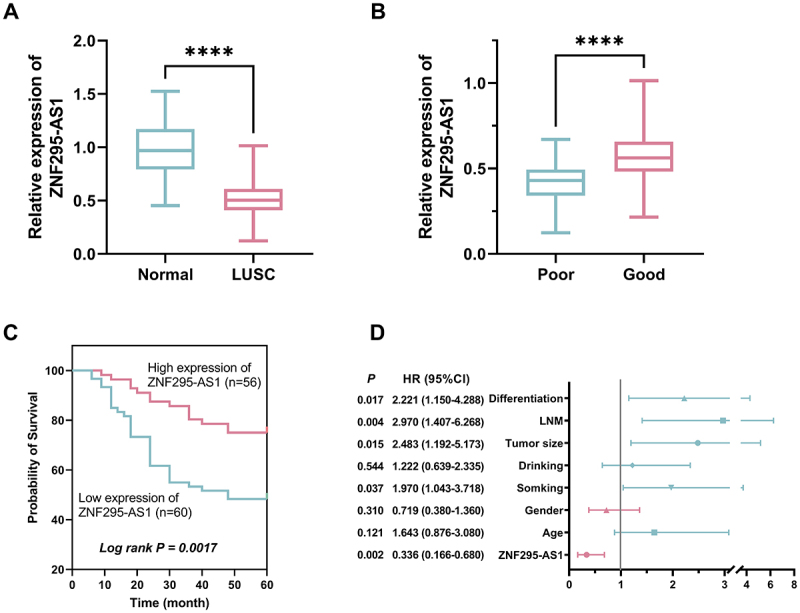
ZNF295-AS1 expression is significantly downregulated in LUSC tissues (A); ZNF295-AS1 levels were lower in LUSC tissues with poor prognosis (B); patients in the ZNF295-AS1 low-expression group exhibited lower survival rates (C); ZNF295-AS1, smoking, tumour size, LNM, and differentiation were independent predictors of poor prognosis in LUSC (D, ***p < 0.001 vs normal or poor). Note: all data were normalized using the normal group as the reference.

Compared with normal tissue, ZNF295-AS1 level was notably downregulated in LUSC tissue (Figure 1(A), *p* < 0.001). Furthermore, ZNF295-AS1 expression levels were lower in LUSC patients with poor prognosis than in those with a favourable prognosis (Figure 1(B), *p* < 0.001).

Furthermore, patients were stratified into high-level and low-level groups based on ZNF295-AS1 levels. Clinical characteristics were compared between the two groups. Results showed no significant differences in age, gender, or alcohol consumption (*p* > 0.05). Significant differences were observed in smoking status, tumour size, lymph node metastasis (LNM), and tumour differentiation (Table 1, *p* < 0.05). Kaplan-Meier curves analysed survival outcomes for both groups. Results demonstrated lower survival rates in the ZNF295-AS1 low-level group (Figure 1(C), log-rank *p* = 0.0017). The results of the Cox regression analysis are shown in Figure 1(D). ZNF295-AS1 expression was identified as a protective factor for LUSC prognosis, while smoking, tumour size, LNM, and differentiation were determined to be risk factors for poor prognosis.

**Table 1.** Correlation between ZNF295-AS1 levels and clinical features in patients with LUSC.

Parameters	ZNF295-AS1 expression		*P*
	Low (*n* = 60)	High (*n* = 56)	
Age, years			
≤60	33	28	0.590
>60	27	28	
Gender			
Male	22	24	0.496
Female	38	32	
Smoking			
NO	28	37	0.035
YES	32	19	
Drinking			
NO	32	28	0.720
YES	28	28	
Tumour size, cm			
≤5	28	42	0.002
>5	32	14	
LNM			
NO	23	35	0.009
YES	37	21	
Differentiation			
well, moderate	25	37	0.008
poor	35	19	

LUSC, Lung squamous cell carcinoma; LNM, Lymph Node Metastasis.

### Effects of ZNF295-AS1 on the LUSC cell line

**Figure 2.**
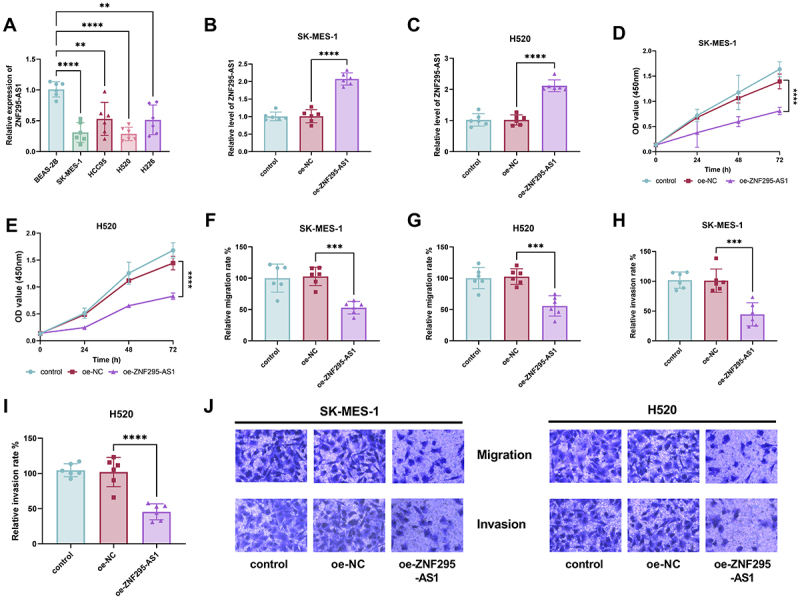
Compared with BEAS-2B cells, the expression levels of ZNF295-AS1 were significantly reduced in the four LUSC cell lines (A); Following transfection to overexpress ZNF295-AS1, its expression levels significantly increased in the cells (B-C); elevated ZNF295-AS1 levels markedly suppressed the proliferative capacity of LUSC cell lines (D-E); and upon ZNF295-AS1 upregulation, the migration and invasive capabilities of LUSC cell lines were also significantly reduced (F-I); images of cell migration and invasion confirm these changes (J, *p < 0.01, **p < 0.001 vs BEAS-2B or oe-NC).

ZNF295-AS1 level was detected in five cell lines. Compared to BEAS-2B, ZNF295-AS1 expression levels were notably reduced in four LUSC cell lines (Figure 2(A), *p* < 0.01). Notably, ZNF295-AS1 expression decreased more markedly in SK-MES-1 and H520. Therefore, these two cell lines were selected for subsequent studies. Following transfection to overexpress ZNF295-AS1, its expression levels significantly increased in two cells (Figure 2(B,C), *p* < 0.001). Elevated ZNF295-AS1 levels markedly reduced the proliferative capacity of two cells (Figure 2(D,E), *p* < 0.001). The migration and invasion capabilities of the two cells were also notably reduced following ZNF295-AS1 upregulation (Figure 2(F–I), *p* < 0.001). Cell migration and invasion images further corroborated these trends (Figure 2(J)).

### Targeted binding of ZNF295-AS1 to miR-96-5p

**Figure 3.**
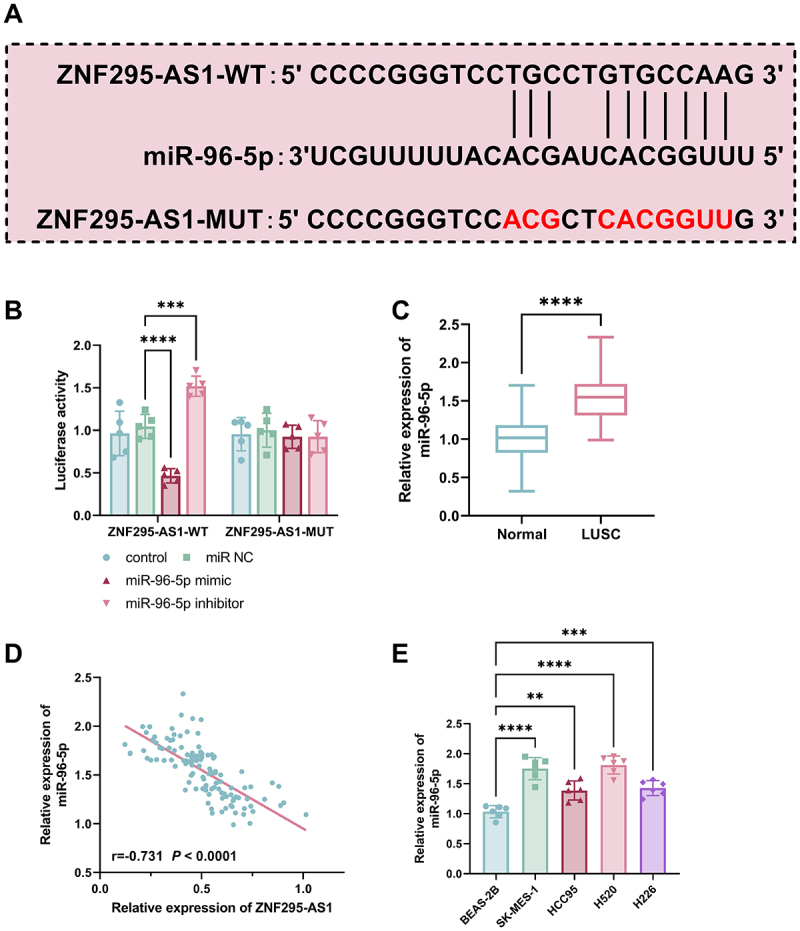
Predicted binding site between ZNF295-AS1 and miR-96-5p (A); dlr experiments validated the binding relationship between ZNF295-AS1 and miR-96-5p (B); Compared with normal tissue, miR-96-5p expression levels were significantly elevated in LUSC tissue (C); ZNF295-AS1 and miR-96-5p expression showed a negative correlation (D); Compared with BEAS-2B cells, miR-96-5p expression was significantly increased in four LUSC cell lines (E, *p < 0.01, **p < 0.001 vs control, BEAS-2B or normal).

Figure 3(A) shows the predicted binding site between ZNF295-AS1 and miR-96-5p from the lncRNASNP2 database. DLR results validated the binding relationship between ZNF295-AS1 and miR-96-5p. Following transfection with miR-96-5p mimics, the luciferase activity of ZNF295-AS1-WT was significantly reduced; conversely, transfection with miR-96-5p inhibitors markedly increased ZNF295-AS1-WT luciferase activity. However, this treatment did not affect the enzyme activity of ZNF295-AS1-MUT (Figure 3(B), *p* < 0.001). This confirmed the specific targeting relationship between ZNF295-AS1 and miR-96-5p.

Compared to normal tissue, miR-96-5p expression levels were significantly elevated in LUSC tissue (Figure 3(C), *p* < 0.001). This indicates that miR-96-5p functions as an oncogenic factor in LUSC. This finding is consistent with the results presented in Table S1 and Figure S1. miR-96-5p was significantly correlated with LNM and tumour differentiation grade, and served as a risk factor for poor prognosis in LUSC patients. Crucially, Pearson correlation analysis revealed a typical negative correlation between ZNF295-AS1 expression and miR-96-5p expression (Figure 3(D), *r* = −0.731, *p* < 0.0001). Compared to BEAS-2B, miR-96-5p level was notably elevated in LUSC cell lines, with the most pronounced increase observed in SK-MES-1 and H520 cells (Figure 3(E), *p* < 0.01).

### ZNF295-AS1 and miR-96-5p together affect LUSC cells

**Figure 4.**
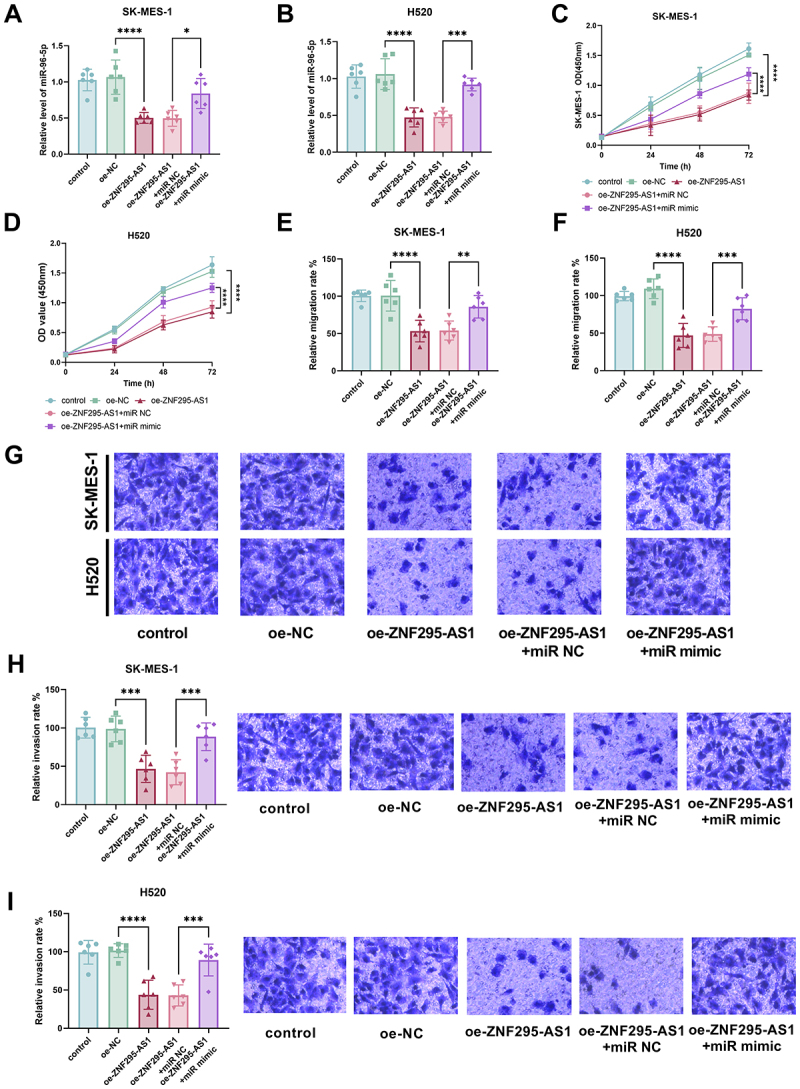
Following transfection with ZNF295-AS1 overexpression, miR-96-5p levels in cells significantly decreased. Conversely, transfection with miR-96-5p mimics markedly elevated intracellular miR-96-5p levels (A-B); when miR-96-5p levels were reduced, the proliferative capacity of LUSC cells was significantly impaired; conversely, increasing miR-96-5p levels reversed this effect (C-D). Following miR-96-5p upregulation, the impaired migration and invasion capabilities of LUSC cells were significantly restored, with cell migration and invasion images confirming this change (E-I, *p < 0.01, **p < 0.001 vs oe-NC or oe-ZNF295-AS1 + miR NC).

Following transfection with ZNF295-AS1 overexpression, cellular ZNF295-AS1 expression levels increased while miR-96-5p levels decreased notably. Conversely, transfection with miR-96-5p mimics led to a marked elevation in cellular miR-96-5p levels (Figure 4(A,B), *p* < 0.001). When miR-96-5p levels were reduced, the proliferation capacity of LUSC cell lines significantly decreased; conversely, increasing miR-96-5p levels markedly restored cellular proliferation (Figure 4(C,D), *p* < 0.001). Following miR-96-5p upregulation, the impaired migration capacity of both cell lines was significantly restored (Figure 4(E,F), *p* < 0.001). Migration images further corroborated this trend (Figure 4(G)). Moreover, increasing miR-96-5p levels markedly reversed the cells’ invasive capacity (Figure 4(H,I), *p* < 0.001).

### Downstream target genes of miR-96-5p and their bioinformatics analysis

**Figure 5.**
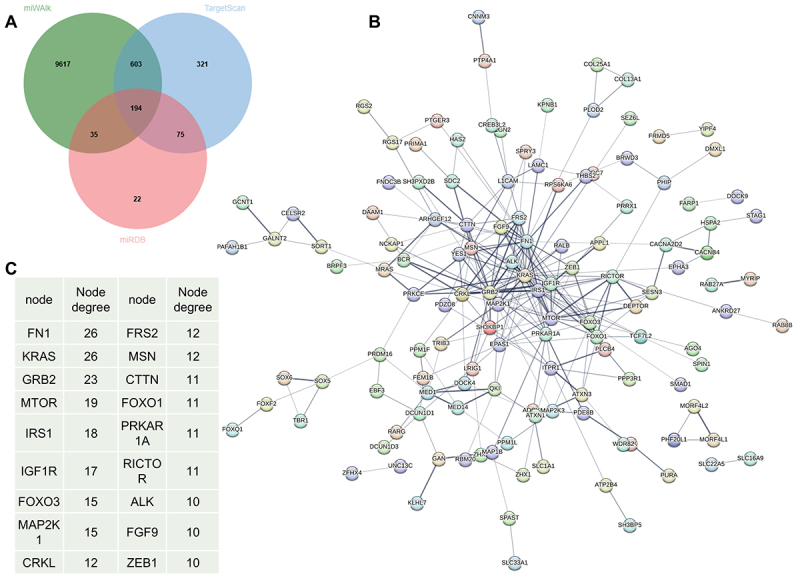
A total of 194 downstream target genes of miR-96-5p were screened using miRwalk, TargetScan, and miRDB databases (A). Protein interaction network analysis results (B); top ten target genes ranked by node degree (C).

A total of 194 downstream target genes of miR-96-5p were screened through the miRWalk, TargetScan, and miRDB databases (Figure 5(A)). The results of the PPI network analysis are shown in Figure 5(B). The PPI network consists of 194 nodes and 278 edges. The average node degree is 2.87. The PPI enrichment *p*-value is less than 1 × 10^−16^. We specifically highlight target genes with a node degree ≥ 10 (Figure 5(C)).

**Figure 6.**
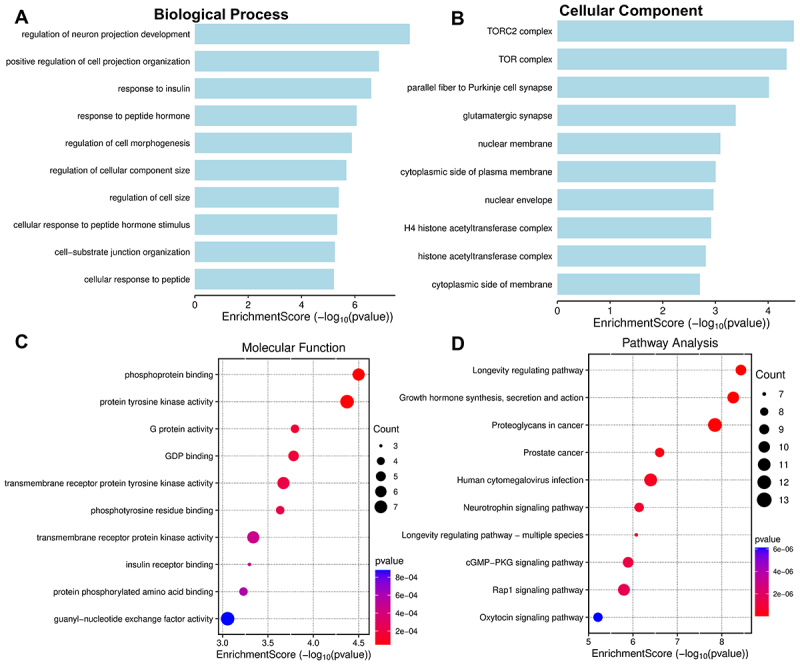
GO data revealed these target genes primarily enriched in biological processes (A); cellular components (B); molecular functions (C); KEGG analysis identified key signalling pathways associated with target genes (D).

Based on PPI results, we selected target genes with node degree > 0 for GO and KEGG analysis. GO data revealed biological process enrichment primarily in the regulation of neuron projection development and positive regulation of cell projection organization (Figure 6(A)). Cellular components were enriched in the TORC2 complex, the TOR complex, and the parallel fibre to Purkinje cell synapse (Figure 6(B)). Molecular functions were enriched in phosphoprotein binding and protein tyrosine kinase activity (Figure 6(C)). KEGG analysis revealed that these target genes were primarily associated with signalling pathways, including Proteoglycans in cancer and Rap1 signalling pathway (Figure 6(D)).

## Discussion

LUSC is a type of non-small cell LC that grows and spreads quickly. Unfortunately, it often does not respond well to treatment [19]. Therefore, identifying novel prognostic biomarkers and therapeutic targets is crucial for improving the clinical management of LUSC. The role of lncRNAs as key regulatory molecules in cancer progression has become increasingly prominent [20,21]. Their abnormal expression in LUSC is closely associated with tumorigenesis, progression, and prognosis. The functions of lncRNAs DUXAP8 [22] and HOTAIR [23] in LC have been extensively studied. In this study, we focus on lncRNA ZNF295-AS1. We systematically investigated the clinical significance and potential regulatory mechanisms of ZNF295-AS1 in LUSC for the first time. Our findings revealed that ZNF295-AS1 was significantly downregulated in both LUSC tissues and cell lines. Furthermore, Kaplan-Meier survival analysis indicated a significant correlation between low ZNF295-AS1 levels and diminished overall survival in LUSC patients. Multivariate Cox regression analysis further confirmed ZNF295-AS1 as an independent prognostic factor for LUSC. Collectively, these findings establish ZNF295-AS1 as a potential valuable prognostic predictor for LUSC.

Extensive research indicates that lncRNAs influence tumour progression by regulating various cell cycle, proliferation, apoptosis and so on [24,25]. To investigate the specific biological function of ZNF295-AS1 in LUSC, we validated it using an *in vitro* cell model. Results revealed that overexpression of ZNF295-AS1 in LUSC cell lines effectively suppressed abnormal proliferation, migration, and invasive capabilities of tumour cells. This suggests ZNF295-AS1 functions as a tumour suppressor gene in LUSC. We hypothesize that its antitumor effect may involve interference with cell cycle progression, thereby blocking rapid expansion of tumour cells.

One of the core mechanisms by which lncRNAs exert their functions is through acting as ceRNAs that bind to miRNAs [26], thereby relieving miRNA suppression of their downstream target genes and forming complex ceRNA regulatory networks [27,28]. For example, the lncRNA NNT-AS1 regulates LC progression via the NNT-AS1/miR-3666/E2F2 axis [29]. LncRNA OIP5-AS1 modulates the biological behaviour of LC cells by regulating the has-miR-29b-3p/ZIC5 axis [30]. This study first validated that ZNF295-AS1 can directly target and bind miR-96-5p, and that ZNF295-AS1 negatively regulates the level of miR-96-5p. Given that miR-96-5p has been extensively reported as an oncogene in multiple cancers, we hypothesize that ZNF295-AS1 May exert its tumour-suppressive function by acting as a ‘sponge’ to sequester miR-96-5p, thereby reducing its levels and inhibiting abnormal proliferation and invasion of tumour cells.

Furthermore, to further investigate the signalling pathways potentially involved in this ceRNA network, we predicted miR-96-5p target genes using multiple databases and performed GO and KEGG enrichment analyses on core target genes. Results showed that these target genes were significantly enriched in biological processes closely related to cancer progression, such as phosphoprotein, protein tyrosine kinase, and proteoglycans in cancer. Numerous studies have demonstrated that protein tyrosine kinase is associated with LC metastasis [31]. For instance, ferritin-tyrosine kinase promotes invasion and tumour metastasis in lung adenocarcinoma cells [32]. Therefore, we hypothesize that ZNF295-AS1 participates in protein tyrosine kinase activity by targeting miR-96-5p, thereby influencing LUSC progression. Furthermore, proteoglycans in cancer can mediate LC cell adhesion [33], correlating with metastasis. Thus, we hypothesize that ZNF295-AS1 and miR-96-5p may also influence LUSC progression and metastasis by affecting proteoglycans in cancer.

Of course, this study has several limitations. First, the specific downstream target genes regulated by the ZNF295-AS1/miR-96-5p axis have not yet been experimentally validated one by one. Second, ZNF295-AS1 May exert its oncogenic effects through alternative mechanisms or miRNAs, such as interacting with RNA-binding proteins or regulating chromatin accessibility. Finally, the biological functions of this axis require validation in additional models, including animal models. Future investigations into these aspects will contribute to further elucidating the regulatory mechanisms of the ZNF295-AS1/miR-96-5p axis in LUSC.

In summary, this study systematically reveals for the first time the clinical value and potential regulatory mechanisms of lncRNA ZNF295-AS1 in LUSC. We confirmed the downregulation of ZNF295-AS1 in LUSC tissues and cells, with its reduced expression correlated to poor patient prognosis. Elevating ZNF295-AS1 levels by directly targeting miR-96-5p downregulation can influence LUSC cell proliferation and invasion, thereby mitigating disease progression and improving patient outcomes. Our work not only provides a novel biomarker for LUSC prognosis prediction but also establishes the theoretical basis for positioning the ZNF295-AS1/miR-96-5p axis as a future therapeutic target in LUSC treatment.

## Supplementary Material

Supplemental Material

Supplemental Material

## Data Availability

The datasets used and/or analysed during the current study are available from the corresponding author on reasonable request.
