# Supplementary material for: The lncRNA ZNF295-AS1 alleviates lung squamous cell carcinoma progression by reducing miR-96-5p and inhibiting cancer cell invasiveness
Source: RNA Biol. 2026 May 14;23(1):1–12. doi: 10.1080/15476286.2026.2669707 (PMC13215284; doi:10.1080/15476286.2026.2669707)
Supplement: Supplemental Material [file KRNB_A_2669707_SM4959.docx]

**Tale S1** Correlation between miR-96-5p levels and clinical features in patients with LUSC

| Parameters | miR-96-5p expression | | *P* |
| --- | --- | --- | --- |
|  | Low (n = 58) | High (n = 58) |  |
| Age, years |  |  |  |
| ≤ 60 | 30 | 31 | 0.852 |
| > 60 | 28 | 37 |  |
| Gender |  |  |  |
| Male | 22 | 24 | 0.704 |
| Female | 36 | 34 |  |
| Smoking |  |  |  |
| NO | 37 | 28 | 0.092 |
| YES | 21 | 30 |  |
| Drinking |  |  |  |
| NO | 29 | 31 | 0.710 |
| YES | 29 | 27 |  |
| Tumor size, cm |  |  |  |
| ≤ 5 | 41 | 29 | 0.023 |
| > 5 | 17 | 29 |  |
| LNM |  |  |  |
| NO | 35 | 23 | 0.026 |
| YES | 23 | 35 |  |
| Differentiation |  |  |  |
| well, moderate | 37 | 25 | 0.026 |
| poor | 21 | 33 |  |

LUSC, Lung squamous cell carcinoma; LNM, Lymph Node Metastasis.
