# Supplementary figures and images for: The lncRNA ZNF295-AS1 alleviates lung squamous cell carcinoma progression by reducing miR-96-5p and inhibiting cancer cell invasiveness
Source: RNA Biol. 2026 May 14;23(1):1–12. doi: 10.1080/15476286.2026.2669707 (PMC13215284; doi:10.1080/15476286.2026.2669707)

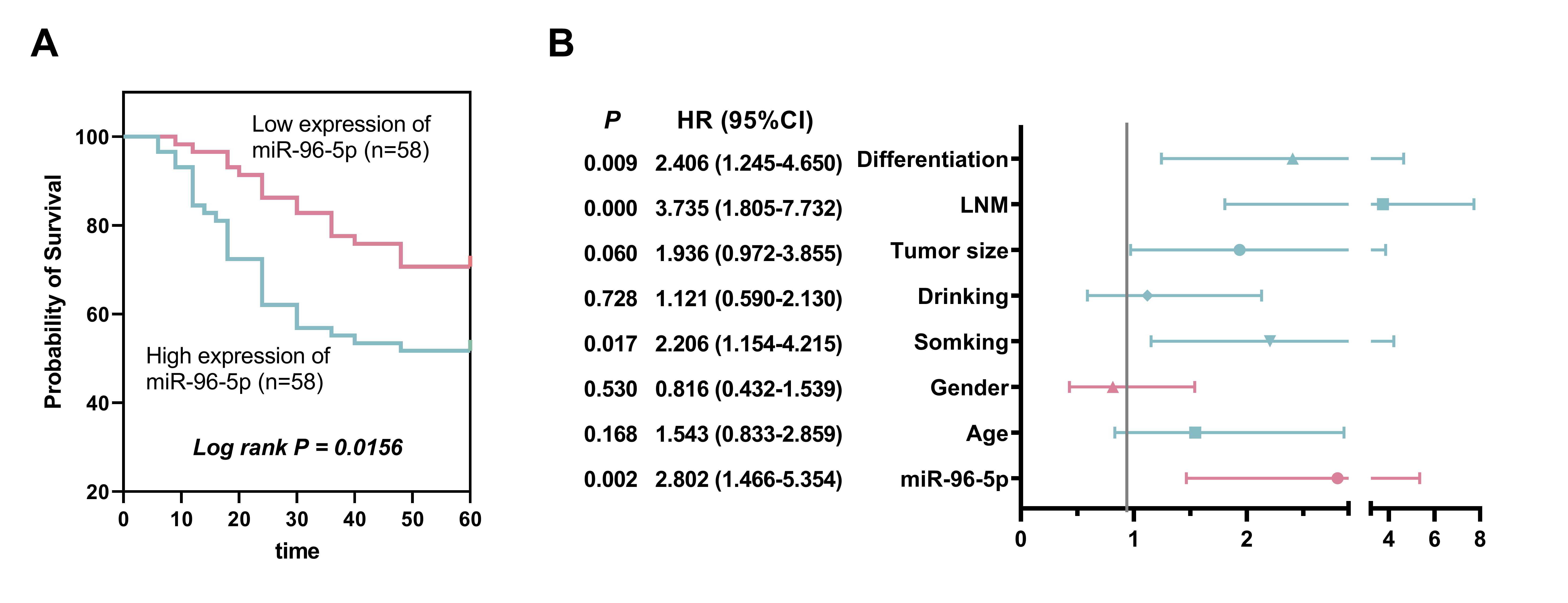

Supplement: Supplemental Material [file KRNB_A_2669707_SM4958.tif]
